# Experiences of people living with HIV and people living close to them of a comprehensive HIV stigma reduction community intervention in an urban and a rural setting

**DOI:** 10.1080/17290376.2014.938104

**Published:** 2014-07-14

**Authors:** Heleen French, Minrie Greeff, Martha J. Watson

**Affiliations:** ^a^ M.Cur, Doctoral candidate, Ms at the Africa Unit for Transdisciplinary Health Research (AUTHeR), Faculty of Health Sciences, North-West University, Potchefstroom Campus, Potchefstroom, South Africa; ^b^ M.Cur, PhD, Professor at the Africa Unit for Transdisciplinary Health Research (AUTHeR), Faculty of Health Sciences, North-West University, Potchefstroom Campus, Potchefstroom, South Africa; ^c^ M.Cur, PhD, Dr at the School of Nursing Sciences, Faculty of Health Sciences, North-West University, Potchefstroom Campus, Potchefstroom, South Africa

**Keywords:** community, comprehensive, HIV, intervention, stigma, communauté, global(e), VIH, intervention, stigmatisation

## Abstract

HIV stigma remains high globally. Although there is a selection of HIV stigma reduction interventions discussed in the literature, there is a paucity of research about the effectiveness of these interventions. This study aimed at gaining a deeper understanding of the experiences of people living with HIV (PLWH) and people living close to them from six designated groups during and after having undergone a comprehensive HIV stigma reduction community intervention in both an urban and a rural setting. Attention was focused on their expressed experiences of the workshop and projects executed. A qualitative interpretive description approach was used. PLWH as participants were selected through purposive voluntary sampling and through snowball sampling for the people living close to them. Recruitment was from both urban and rural settings in the North West Province, South Africa. Data collection was via in-depth interviews with 23 PLWH and 60 people living close to them from specific designated groups. The data were thematically analysed through manual open coding. The results from the urban and rural settings were pooled, as there were no noteworthy differences in the themes between them. The results indicated that there was an increase in knowledge in all the groups, as well as experiences of enhanced relationships and of being equipped with leadership skills in order to go out into the community and being part of HIV stigma reduction actions. The intervention in its comprehensive nature was found to have been successful and promising for future use in reducing HIV stigma.

## Résumé

La stigmatisation liée au VIH est répandue dans le monde. Bien que la documentation sur le sujet aborde un ensemble d'interventions en faveur de sa réduction, les recherches sur l'efficacité de telles interventions restent limitées. Cette étude vise donc à mieux comprendre l'expérience des personnes vivant avec le VIH (PVVIH) et de leur entourage dans six groupes sélectionnés pendant et après une intervention communautaire globale de réduction de la stigmatisation liée au VIH, en milieu urbain et rural. L'on a accordé une attention particulière aux expériences partagées sur l'atelier et les projets entrepris. Une approche de description qualitative et interprétative a été adoptée. Les PVVIH participantes ont été sélectionnées grâce à un échantillonnage dirigé et volontaire, et un échantillon boule de neige pour l'entourage. Les personnes recrutées provenaient de milieux urbains et ruraux de la province du Nord-Ouest, Afrique du Sud. La collecte de données s'est faite par des entretiens approfondis avec 23 PVVIH et 60 personnes de leur entourage, toutes issues de groupes spécifiques désignés. Les données ont été analysées thématiquement par codage ouvert et manuel. Les résultats des milieux urbains et ruraux ont ensuite été regroupés car il ne semblait pas y avoir de différence remarquable entre les thèmes soulevés dans les deux milieux. Dans tous les groupes, les résultats indiquaient un accroissement des connaissances, de l'amélioration des relations, et des compétences de leadership pour prendre part à la vie communautaire et participer aux activités de réduction de la stigmatisation. La réussite de la nature globale de l'intervention est prometteuse pour les activités futures de réduction de la stigmatisation du VIH.

## 1. Introduction and problem statement

This article specifically reports on the in-depth experiences of people living with HIV (PLWH) and people living close to them of a comprehensive HIV stigma reduction community intervention, and not the intervention itself. People living close to them comprised partners, children, close family members, friends, spiritual leaders and community members. The research forms part of a larger SANPAD-funded study focusing on HIV stigma reduction in both an urban and a rural setting in the North West Province, South Africa.

South Africa is known to be the country with the highest HIV rates globally, but fortunately there is evidence that its incidence is decreasing. The persistency of the disease is, however, challenged by current advances in HIV medicine and the free access to antiretroviral treatment (Department of Health 2011; South Africa.info 2013). These advances make HIV a manageable condition in the long term. However, the stigma attached to HIV remains a major problem, mainly due to the probability of immoral behaviour associated with its cause (De Bruyn 1999; Pape 2005). This perception has led to a great number of people being negatively affected by HIV stigma.

A group of researchers (Holzemer, Uys, Makoae, Stewart, Phetlhu, Dlamini, *et al*. 2007; Uys, Chirwa, Kohi, Greeff, Naidoo, Makoae, *et al*. 2009) conducted intensive research on HIV stigma within the African setting over a five-year period. They aimed to understand HIV stigma within the African setting, formulating a conceptual model for HIV stigma, and developing and validating two stigma scales for the African context (Holzemer *et al*. 2007). Their study also had a component focusing on HIV stigma reduction in healthcare settings, including both PLWH and nurses (Uys *et al*. 2009). The current study proposed to extend this previous research into the community.

According to the conceptual model of HIV stigma in five African countries, stigma is a complex process and it occurs within a context consisting of the environment, the healthcare system and different agents. The stigma process itself involves triggers of stigma, stigmatising behaviours, types of stigma and outcomes of stigma (Holzemer *et al*. 2007). Stigmatising behaviour comprises discriminatory acts towards PLWH in different degrees. The types of stigma identified in the conceptual model include received stigma, internal stigma and associated stigma. This model formed the theoretical framework for the current study. The definition of HIV stigma as compiled by Alonzo and Reynolds (1995, p. 304) is supported in this study. They describe stigma as ‘a powerful discrediting and tainting social label that radically changes the way individuals view themselves and are viewed as persons’.

Despite the negative impact on PLWH and people living close to them, there have been a surprisingly limited number of intervention studies aimed at reducing HIV stigma. The most prominent HIV stigma reduction studies as summarised in recent literature reviews by various authors mainly focused only on PLWH (Brown, Macintyre & Trujillo 2003; Heijnders & Van der Meij 2006; Sengupta, Banks, Jonas, Miles & Smith 2011). Brown *et al*. (2003) reviewed 22 studies that evaluated HIV stigma reduction interventions. They described them as being one of four types: information-based approaches, skills building, counselling approaches and improvement of contact with the affected group. Results of these interventions indicate that there are some that seem to be effective, at least on a small scale and in the short term, but this is inadequate, especially in relation to the scale and the duration of the impact of stigma reduction.

The review done by Heijnders and Van der Meij (2006) did not focus specifically on HIV and AIDS-related stigma reduction but it was significant, as it described related stigma reduction strategies focusing on individuals at interpersonal as well as community level. Sengupta *et al*. (2011) also did a literature review, focusing on evaluating the effectiveness of HIV stigma reduction interventions in which HIV and AIDS stigma was one of the outcomes measured. Data were extracted from 19 studies, 14 of which demonstrated effectiveness in reducing HIV and AIDS stigma. However, only 2 of these 14 effective studies were considered good studies on the basis of their quality and the extent to which the intervention focused on reducing HIV and AIDS stigma.

The intervention studies were mainly quantitative, and the literature lacks qualitative reflection on experiences of these interventions that could be good for future HIV stigma reduction intervention development. It was further noted that there is a serious need for more comprehensive approaches towards HIV stigma reduction. Some target-specific interventions were found, for example focusing on people, like the designated groups in the current study. Uys *et al*. (2009) executed an HIV stigma reduction intervention focusing on the healthcare setting. The study proved to be successful in that it led to enhancing contact with PLWH, increasing knowledge of HIV and stigma and coping through empowerment. It instigated an increase in voluntary HIV testing by the nurses in the group, and was effective in reducing perceived stigma by PLWH and improving self-esteem (Uys *et al*. 2009).

HIV stigma reduction interventions targeting partners, like in a study by Manyedi, Greeff and Koen (2010), developed a programme to empower women whose partners had died of AIDS to cope with the accompanied stigma. The International Centre for Research on Women (Duvvury, Prasad & Kishore 2006) developed a manual aimed at providing strategies for the reduction of HIV and AIDS stigma and violence against women. Sallar and Somda (2011) suggest a communication strategy such as entertainment education to raise awareness and tolerance and to promote action in the fight against HIV and AIDS stigma towards male partners engaging in sexual activity. It seemed from the literature that stigma reduction interventions targeting the partner mostly focus on disclosure issues and health education approaches with regard to safe sexual practices.

HIV stigma reduction interventions targeting the child also showed to be individual based and in most cases did not include PLWH. The ‘Save the Children’ study in China in 2005, for example, indicated that taking children seriously, having fun with them, treating them with admiration and using group activities were found to provide psycho-social support and promote personal development (Children in Distress Network 2007).

Another example of an HIV stigma reduction intervention is entitled ‘Engaging youth to provide care and tackle stigma in rural Zambia’. The aim of the programme was to involve school-aged children in the care and support of PLWH. The findings showed intensely positive attitude changes by family members and the wider community in general. Benotsch, Seal, Stevenson, Sitzler, Kelly, Bogart, *et al*. (2008) mention that some educational efforts focusing on children and HIV stigma reduction in Africa were undertaken and were effective, as they enhanced knowledge. Their sustainability and effects have not yet been evaluated though.

Fawole *et al.*, as quoted by Benotsch *et al.* (2008), similarly applied educational approaches in the form of six weekly information sessions for secondary school children in Nigeria. Results indicated significant increases in tolerance for PLWH. A television series known as ‘Soul Buddyz’ was found to be a rewarding production for children, as they talked about things that they had seen on ‘Soul Buddyz’ (UNAIDS 2005). It seems as though HIV stigma reduction interventions targeting the child are mainly focusing on education and entertainment, and none were found that involved PLWH.

HIV stigma reduction interventions focusing specifically on family members of PLWH are uncommon in the literature. An example of an intervention aimed at HIV stigma reduction in the community is the ‘FARM project’ (Foundation for Agricultural and Rural Management) in Thailand, which provides training for family members and community members on home-based care that aims to reduce HIV stigma in the family context by opposing myths and reducing cases of isolation (Busza 1999). Another example is the study by Krishna, Bhatti, Chandra and Juvva (2005). These authors attempted to understand the impact that stigma had on the family system, as the family in the Indian context usually represents emotionally strong bonds.

There is a significant gap in the literature regarding family member-oriented HIV stigma reduction interventions in particular. Likewise, HIV stigma reduction interventions with friends are minimal. In no portion of literature the researcher reviewed could any interventions specifically targeting the ‘friend’ of PLWH be found. This emphasises the significance of this comprehensive HIV stigma reduction intervention.

Nyblade, Stangl, Weiss and Ashburn (2009) describe an HIV stigma reduction intervention that was implemented in two urban communities in Vietnam that was attended by several individuals, some of whom knew each other, for example as neighbours. The main aim of the intervention was to increase awareness of HIV stigma and reduce fear-driven stigma, value-driven stigma and discrimination. Qualitative studies depicting experiences of HIV stigma reduction programmes are scarce. The literature review also suggests that there is no comprehensive approach towards stigma reduction.

Limited HIV stigma reduction studies focusing on spiritual leaders were found in the literature. An HIV stigma reduction programme by the Malaysian AIDS Council managed to gain support from the Minister for Islamic Affairs (UNAIDS 2005). The religious authorities of nine out of 14 states attended the initial workshop. The SanghaMetta (‘Compassionate Brethren’) project was introduced by a lay Buddhist teacher in Northern Thailand in 1997. The aim was to make use of existing community resources and extend the traditional role that Buddhist monks and nuns play in social welfare in the region towards HIV prevention and care. The SanghaMetta training model was also used effectively with Christian, Hindu and Islamic leaders from Sri Lanka, Nepal, Pakistan and Afghanistan (UNAIDS 2005). The outcomes of these interventions mentioned were education-focused and partially assisted in stigma reduction, as they enhanced disclosure and also assisted in reducing fear of interacting with PLWH.

HIV stigma reduction interventions focusing on the community as a whole were generally found in the form of outreach activities aimed at demonstrating the low risk associated with caring for PLWH. In Cambodia, for example, there are home-visit care teams that visit suspected PLWH weekly and then train their primary caregivers via demonstration that close contact does not cause HIV transmission (Busza 1999).

In a study by Watson (2008), a community-based collaboration to support the older person in the world of HIV and AIDS, the author focused on this vulnerable group (previously disadvantaged elderly community) affected by HIV and AIDS. Although the main aim of her research was not merely to reduce HIV and AIDS stigma, she certainly addressed this problem as being part of the detraction from their overall well-being.

A community participation intervention for the reduction of HIV-related stigma in Thailand (Apinundecha, Laohasiriwong, Cameron & Lim 2007) improved the levels of accurate HIV and AIDS knowledge among participants and also reduced the level of community stigma in the intervention group compared to the control group. The Academy for Educational Development [AED] (2007) designed a toolkit for action against HIV stigma. It was designed by and for HIV trainers in Africa to assist them in the planning and organisation of educational sessions to raise awareness and promote practical action to challenge HIV stigma and discrimination. It is an elementary, comprehensive educational tool for the lay person in the general community (AED 2007).

Numerous community-based interventions with multiple activities demonstrated substantial changes in stigma at community level in Thailand, Tanzania, Vietnam and Zambia. Each of these programmes focused on community participation. Intervention studies in Vietnam and Tanzania suggested that opportunities for ongoing discussions about values and beliefs were imperative for reducing more than fear-based stigma and combating other drivers of stigma (Duvvury *et al*. 2006; UNAIDS 2009).

The literature on HIV stigma reduction interventions targeting specific groups of people living close to PLWH strongly suggests that the increasing of knowledge is the main objective. There is, however, a need to move outside information and education approaches and to rather integrate the necessary elements for an effective response to sustainable stigma reduction outcomes (Eba 2007). There is furthermore a paucity of research on the experiences of the participants after they had undergone HIV stigma reduction interventions.

Authors also seem to come to opposing conclusions with regard to HIV stigma manifestations in urban and rural contexts, making effective intervention planning difficult. Naidoo, Uys, Greeff, Holzemer, Makoae, Dlamini, *et al*. (2007) found that PLWH from urban contexts were often being stigmatised more than their rural equivalents. However, there are studies that found that the opposite was true (Bunn, Solomon, Varni, Miller, Forehand & Ashikaga 2008; Heckman, Somlai, Kalichman, Franzoi & Kelly 1998). It is argued that the social background of the people involved as well as their economic status may influence their way of comprehending HIV, and hence their behaviour with respect to stigmatisation and discrimination.

From the literature reviewed, it was evident that there are gaps in scientifically based HIV stigma reduction interventions aimed at curbing the phenomenon on a long-term basis (Sengupta *et al*. 2011). There are insufficient HIV and AIDS stigma reduction interventions, measurement tools evaluating the effects of HIV stigma reduction interventions are lacking and the impact of the interventions on public health is not taken into consideration. It is important to provide proof of whether a reduction in HIV and AIDS stigma is associated with better health outcomes. It is therefore important to look into the experiences and outcomes of HIV stigma reduction interventions in order to improve future interventions. It is also useful to see whether there are any differences in HIV stigma reduction experiences between urban and rural contexts.

## 2. Research objective

In this study the aim was to compare the expressed experiences of PLWH and people living close to them in an urban and a rural setting after they had undergone a comprehensive HIV stigma reduction community intervention.

## 3. Research design

The research followed a qualitative interpretive description approach (Thorne 2008) in order to explore and describe the expressed experiences of PLWH and people living close to them after they had undergone a comprehensive HIV stigma reduction community intervention. The research took place in urban Potchefstroom and rural Ganyesa. Both settings mainly included individuals from an African Setswana background. The living conditions in these areas are mainly poverty driven due to high rates of unemployment (North West Provincial Government 2013).

## 4. Research method

This is a novice approach to HIV stigma intervention including PLWH and all the various groups of people living close to them. This intervention involves PLWH and the people living close to them at the same time in a group.

### 4.1. Sample

The sample was drawn from two main groups, namely PLWH and people living close to them originating from the urban greater Potchefstroom and the rural Ganyesa in the North West Province, South Africa. PLWH were gathered by means of purposive voluntary sampling (Burns & Grove 2005). PLWH were recruited through mediators with longstanding trust relationships with PLWH from local healthcare facilities and non-governmental organisations. The research assistant was informed of the willing participants and given their contact information. For PLWH, the inclusion criteria were that they had to have been diagnosed with HIV for at least six months and they had to be able to communicate freely in English, Afrikaans or Setswana; willing to take part in a study in which HIV-status disclosure is imminent and give consent to be interviewed and recorded. These PLWH were all actively involved in the workshops as well as projects with their designated groups. The final sample size of PLWH was *n* = 18.

The second main group, namely the people living close to them, consisted of six designated groups for each of the separate workshops with them and the PLWH, namely partners, children, close family members, close friends, spiritual leaders and community members of the PLWH. They were gathered through snowball sampling (Strydom 2005). Each of the PLWH was asked to identify appropriate participants of their choice. The inclusion criteria for all six people-living-close-to-them groups were that they had to be at least 18 years of age; able to communicate freely in English, Afrikaans or Setswana and give consent to be interviewed and recorded. Each designated group also had some specific inclusion criteria: namely, the partner had to be married to or had to have been in a stable relationship with the PLWH for a period of at least six months; the child had to be a biological child of the PLWH and be at least 15 years of age or older, the parent had to give permission to take part in the study, and the children had to provide assent. No more than minimal risk was foreseen for them. The close family member could be any person apart from a partner or child. The close friend had to have been in a friendship relationship with the PLWH for a period of at least six months. The spiritual leader could be a traditional healer or religious leader. The community member had to be a person with whom the PWLH had regular contact, such as a neighbour. These people all had to actively participate in the entire intervention. The sample size for the people living close to them was *n* = 60, comprising the six designated groups.

### 4.2. Data collection

The research project was approved by the Research Ethics Committee of the North-West University (NWU-00011-09-A1) and the local Department of Health. For this study all participants were already involved in a larger SANPAD-funded study and have given their consent before. Participants were provided with detailed verbal as well as written information regarding the project and of what would be expected of them. Consent was re-confirmed and documents signed before participation, and they were informed that participation is voluntary and that they could withdraw at any stage.

The researcher initially gained access to the community through a mediator and a research assistant. The research assistant made appointments with each potential participant and informed them of the arranged date, time and venue of interviews. They were thoroughly prepared for the interviews, and allowed time for queries prior to starting. They were assured of confidentiality and anonymity. All documents were kept in locked cabinets and were accessible only by researchers who were directly involved (Burns & Grove 2005; Polit & Beck 2006).

The participants were made aware that sharing experiences may cause discomfort. Participants were further made aware of the availability of counselling and emotional support afterwards should they need it. Benefits included an opportunity to share their experiences of the intervention. Participation would also assist researchers in executing a comprehensive HIV stigma reduction community intervention.

Two open-ended questions for use in the in-depth interviews with PLWH as well as people living close to them were formulated beforehand and evaluated by experts for appropriateness. The questions were further assessed by conducting an interview and were then included in the data set. The PLWH were asked to respond to the following two open-ended questions: ‘How did you experience the workshop and project with people living close to you and others in the group?’ and ‘How did you feel about telling others your HIV-positive status during the workshop and project?’ Two open-ended questions were asked to the various groups of people living close to them: ‘How did you experience the workshop and project with the PLWH and others in the group?’ and ‘How did you feel about hearing the PLWH telling you and others of their HIV-positive status during the workshop and project?’

The interviews individually took around one to one-and-a-half hours to complete. Various communication techniques such as the use of minimal verbal responses, paraphrasing, reflection, clarification, probing and making use of summarisation were utilised. In-depth field notes were made after the interviews (observational, theoretical, methodological and personal notes) for future reference and verification of the process and findings (Botma, Greeff, Mulaudzi & Wright 2010; Greeff 2005).

The intervention for PLWH and people living close to them was adapted from the work done by Uys *et al*. (2009). The intervention consisted of an initial two-day workshop with only the PLWH, in both an urban and a rural setting, focusing on understanding HIV stigma, identifying their personal strengths and training in responsible disclosure management to empower them and ensure their ability to handle disclosure (should they feel the need) in a responsible manner in order to prepare them thoroughly on a psychological level for the next phase of the intervention involving various people living close to them.

This was followed by six three-day workshops in both settings with PLWH and a specific designated group, namely partners, children, close family members, close friends, spiritual leaders and community members. Each group had a project running over a period of a month. The workshops were presented in the form of presentations and small-group discussions and activities.

The facilitators of the workshops were always a non-infected as well as an infected individual. The researchers who acted as presenters during the intervention received in-depth training on the presentation of the course material beforehand. The tenets that these workshops were built upon were increased knowledge and understanding of HIV stigma, equalising the relationship between PLWH and people living close to them and empowering them to handle HIV stigma and enhance their wellness. The workshops aimed at bringing PLWH and people living close to them together, building relationships between them and providing them with knowledge.

The first day of the workshop focused on understanding HIV stigma and coping with it. The second day of the workshop aimed at motivating PLWH to use the knowledge gained on the first day to move into action and develop skills to become leaders in HIV stigma reduction in their own community. Participants were taught how to plan a project, followed by them planning their own projects with designated groups similar to theirs, e.g. partners with partners. PLWH spontaneously formed part of each of the six groups of PLC.

There was a period of one month after the initial two-day workshop for the implementation of the various projects. The researcher facilitated all groups by appointment in order to support them and to monitor their progress.

The third and last day of the workshop took place one month after the initial two-day workshop and each group had the opportunity to give feedback on their executed projects. They had to give a summary of the outcomes of their projects. Evaluation of the projects was done by a panel made up of invited relevant stakeholders in the community and the researchers involved. The evaluation of the projects was based on the feedback by researchers during facilitation as well as the presentation by participants. Feedback was then given to participants by the panel on their successes and a prize was awarded to the best project in the urban as well as the rural setting. The 18 PLWH and 60 people living close to them reached 1793 people with their projects.

### 4.3. Data analysis

Digital voice-recorded interviews were accurately transcribed verbatim in order for data analysis to take place (Botma *et al*. 2010; Hek & Moule 2006). Data analysis was done manually by means of Creswell's generic qualitative analysis approach, which was thematically focused (Creswell 2009). The steps taken by the researcher involved reading the data and making a detailed analysis, with grouping of themes under major topics, unique topics and leftovers. The data were then assembled according to identified topics and into categories and themes. The data were interpreted and conclusions drawn. Analytical bias was avoided through the use of a co-coder to reach consensus.

## 5. Trustworthiness

The researcher applied Lincoln and Guba's model (in Krefting 1991) to guarantee trustworthiness in this research. *Truth value* was assured by prolonged engagement in the research field during the workshops as well as by conducting the in-depth interviews. Reflexivity was achieved through the writing of comprehensive field notes during and after the interviews. The researcher received in-depth training and did simulated interviews in advance. Triangulation of investigators took place. Regular discussions with study leaders took place, improving credibility. *Applicability* was guaranteed by the well-thought through sampling in urban and rural settings, as well as the thick description of the methodology. *Consistency* was ensured in that an audit trail as well as stepwise replication was possible due to a thick description of the methodology. The use of a co-coder further enhanced consistency. *Neutrality* was ensured by the availability of an audit trail, triangulation of investigators and reflexivity.

## 6. Results of the study

The results are based on the in-depth interviews conducted with PLWH as well as the six designated groups who attended the intervention in its totality and took part in the implementation of their HIV stigma reduction projects. See Table 1 for description of the population. During data analysis it was established that there were no major differences in the themes between participants from the urban and rural groups, and data were therefore pooled. If any noteworthy differences are identified, they will be indicated. The experiences of the participants in the comprehensive HIV stigma reduction community intervention, which included the workshops and the projects, will be presented in the following sequence: PLWH, partners, children, family members, friends, spiritual leaders and lastly community members. The discussion will be enriched by quotes of responses by participants. The main aim of reflecting on these experiences is to form an understanding of whether the intervention was successful and to identify gaps for possible improvement for future implementation.

**Table 1.** Sample distribution.

	PLWH						
	Urban			Rural			
	Female	Male	Subtotal	Female	Male	Subtotal	Total
	9	1	10	5	3	8	18
	PLC						
	Urban			Rural			
	Female	Male	Subtotal	Female	Male	Subtotal	Total
Partners	0	2	2	1	0	1	3
Children	3	1	4	5	2	7	11
Family	2	0	2	4	1	5	7
Friend	2	0	2	6	0	6	8
Spiritual leader	2	4	6	7	3	10	16
Community member	6	1	7	8	0	8	15
Total	15	8	23	31	6	37	60

### 6.1. Experiences of PLWH

The PLWH with each of their designated groups uniformly expressed a strong sense of a shift in perception regarding HIV stigma experiences. They became enlightened by the fact that they could, with the support of people living close to them, live in a positive manner with HIV: ‘When I was with the partners, I was able to talk to them about HIV. Before you stigmatise, think first about our life;’ ‘At the workshop I experienced when a person lives with that illness and you have a partner don't point fingers and say it's their fault; … you should come together and fight this together.’

Most of them reported feeling out of place and unsure initially, especially due to the immanent disclosure that was to take place: ‘I felt lonely;’ ‘I was so afraid. So scared to talk about it.’ PLWH felt empowered regarding disclosure choices: ‘ … an eye opener, it teaches me a lot. Because I learned how to disclose my status and how to trust people I disclose to.’

The eventual outcome for most of the PLWH was that of freedom after disclosure and acceptance and comfort received during the intervention. PLWH experienced strengthening of relationships during the intervention. They felt supported and their human dignity enhanced during the intervention: ‘We didn't judge each other, and there was openness. It felt like a blanket that keeps you warm.’ Disclosure brought along a sense of self-determination and freedom: ‘I feel free. And more appreciative:’ ‘I shouldn't hide my status, I'm free;’ ‘I was free. I was confident to disclose myself’. ‘It means that after we attended the workshop I learned a lot, so I'm not afraid to talk about it.’

The PLWH, like all the other designated groups that were part of the intervention, described gaining important knowledge regarding HIV stigma and coping with it. ‘In the workshop I experienced how to cope and what is stigma;’ ‘At first … I didn't understand what the meaning of stigma is. When I came to the group, I learned more about the stigma.’ They described a sense of realisation of how the community was discriminating against them: ‘ … we get undermined and discriminated against by the community;’ ‘They undermine us, and this results in us being side lined.’ PLWH were reminded of the severe emotional pain that was caused by HIV stigma towards them: ‘I was feeling very, very sad and lonely, my family doesn't accept me because I'm HIV positive;’ ‘My sister used to beat me.’

PLWH felt enabled to take part in HIV stigma reduction in their community. They reported that designing a project was not always an easy task, but by joining hands the fight can be successful: ‘It was difficult to plan and organise all of those things, but the support you get from your group members it's what count. That gives you strength to do what you planned to do.’ A strong sense of pride about being part of the intervention was also reported: ‘I felt so proud to be part of the project.’ They expressed their desire to reach more people so that HIV stigma reduction can be an active reality within the broader community: ‘If we can reach more people, stigma will reduce in the community, people will rush to the clinics, they will not fear to go to the clinics because of stigma.’

### 6.2. Experiences of partners

Partners gained knowledge about HIV stigma and they became strongly aware of their own role in stigmatising PLWH: ‘Like I didn't understand that these things of pointing fingers at people with HIV or going around talking about them that they have HIV or AIDS I didn't realise that doing that is stigmatising a person.’ Partners reported a strong sense of learning how to cope with stigma: ‘Like it taught me to accept myself and whatever obstacles and challenges that come my way to be able to face them’. They became empowered to share the knowledge as leaders in HIV stigma reduction in their own community. ‘Because I saw that it does happen to other people in the community. When a person sees that his wife is sick then he blames her, so I was able to talk to my neighbours and guide them’; ‘I am able to encourage people to go and test and share my knowledge with them.’

Partners expressed a sense being united during the workshops and the projects with the goal of reducing stigma: ‘I experienced that we got together, the community as well as PLWH, and we got to teach them about stigma and that PLWH are just like any other person who is living with any other illness’; ‘The project taught me to interact with people and to come together as a group and talk about this illness as partners and not fear anything.’

Partners felt empowered to disclose their own HIV-positive status after observing partners in the group doing so. ‘Sometimes when I was around others and they talked about people with AIDS I used to be ashamed and leave the room but now I am able to talk to them and tell them that we can live with AIDS and take our medication’; ‘I would fear being excluded because I had AIDS so I wouldn't tell people. I couldn't express my feelings but after the workshop I am able to talk to them and tell them that I am still living.’

Partners felt enlightened, enthusiastic and proud to go out and share their knowledge gained. ‘I felt like I could go around and teach other people so that they can also live good lives’; ‘So that the knowledge can move forward’; ‘I was very proud that I got that knowledge to teach the community’. Partners experienced the intervention as an enriching and uplifting experience: ‘It was a life-changing experience and it was the first time hearing about stigma’; ‘My spirit felt supported to hear about stigma’.

They experienced designing a project as challenging yet extremely satisfying and building confidence. ‘Well I didn't even know what a project was. I learnt that if you want to do a project you should work hard.’ ‘I felt like I could in front of a nation and tell them that a person with HIV is also a person’; ‘And I now feel that I am a stronger person and I can tell someone to go forward and tell people about stigmatising.’ Through their group effort they made a success of their projects and felt fulfilled. They learned that group effort ensures success: ‘I learnt that as a group you can be able to reduce stigma as a group if we work together.’

### 6.3. Experiences of children

Children initially felt nervous and unsure of what to expect of the workshops, but as the time went by they reported more self-confidence: ‘I was quite a shy person and I couldn't speak in front of an audience. So, after I attended the workshops that's when I started to feel free’; ‘I learnt to accept yourself.’ People from different age groups were involved in the intervention, which initially made it somewhat intimidating for some children: ‘The workshop was quite scary for me at first because there were older people there’. They also felt scared and unsure of how to react towards PLWH: ‘At first I was scared because I did not know how to react to PLWH.’

However, they got to know the PLWH better and acknowledged a positive kinship between them: ‘We have to embrace the PLWH’. They learned to treat PLWH with dignity and not to discriminate against them: ‘It taught me to accept them and love them and be able to touch them and we can even share a glass.’ Children reported that they had experienced a learning curve during the intervention and that they had gained valuable knowledge throughout: ‘It was kind of a pathway that I had to learn (the project). At some points, I told myself now you need to focus and learn’.

They realised the extent of HIV stigmatisation against PLWH and felt hurt by it. Some felt ashamed as they noticed that they were taking part in stigmatisation: ‘I felt hurt by what these kids were doing because I also taught myself about what stigma was and that how it affected people emotionally and physically’; ‘Because to be honest I was a thrower of rocks, I was one who stigmatised.’

Children noted that people with HIV were often rejected and negative labels were attached to them in their communities: ‘Some people in the community say very bad things about people living with HIV. This makes people living with the disease ashamed.’ The children had a common goal of advocating against HIV stigma: ‘ … because I know my community wasn't aware the stigma attached to HIV. So now they will start being aware.’ ‘These people should be taught that people with HIV are people too.’

They felt that stigma reduction can have a positive outcome for PLWH, as they then did not need to hide from people due to their status: ‘It would really help because people wouldn't have to hide the fact that they have HIV and they would be free.’ They reported forming positive relationships with the other children in the group. The children came to the realisation that there were other children in the community with the same circumstances as themselves. It elicited a sense of belonging and comfort: ‘Knowing other children and understanding how they feel, of being affected by HIV and getting to know their situations at home … ’; ‘We were working like a group and like a family.’

Children reported gaining self-confidence to achieve success through participation: ‘We were working as a team working hard. We told ourselves that we are going to achieve the project.’ They felt excited about completing their project: ‘So we felt great about it.’

### 6.4. Experiences of family members

The group of family members expressed that they got a much better understanding of stigma and that they had not realised what stigmatisation was and how bad it was: ‘I didn't know about this stigma before until I heard all these people;’ ‘I wasn't okay because it was still us who hurt people with our words.’ The family members realised the hurt they caused by stigmatisation and that it was unacceptable and that they should change their behaviour: ‘The workshop and the project made a difference in my life … I learned that I must respect and love people who have been infected with this disease;’ ‘I am no longer going to stigmatise people.’

Leadership was enhanced and stigma activists formed during the intervention: ‘After attending that workshop I can stand in front of the people telling or teaching about stigma and how to handle the people who are living with HIV;’ ‘I learn too many things and it made me feel good because I was a leader … ;’ ‘I feel very happy because right now I can become something. I can teach people what is the stigma.’

The group of family members from the rural setting indicated the presence of blatant discriminatory acts within healthcare settings directed at PLWH. In Ganyesa, almost all participants indicated that they were aware of discrimination at the local healthcare facilities in the form of differentiation by means of colour coding between files of PLWH and people who were not infected: ‘Their files are separate, the colour is not the same as others;’ ‘And I realised that people are afraid of these files because when people see your file that's when they start talking about you.’

The project was described as a difficult task but yet fruitful in the end. Participants from the urban group experienced a challenge regarding commitment, but the group members who persevered succeeded: ‘When the time goes on, the project broke into two pieces;’ In the end they felt proud of succeeding and expressed a sense of achievement: ‘I teach people what is the stigma and understand it very well.’

The rural group of family members also experienced that they had achieved their goals with their project: ‘People are happy about the information and people are promising not to do what they did last.’

### 6.5. Experiences of friends

A strong message of fear of contagion was reported by friends. Some indicated how their distorted ideas had been corrected during the intervention: ‘I was scared of people with HIV. And even to touch them I was scared to touch them. But right now I can touch her; I can do anything for her’. Friends came to realise how painful the effects of stigma were: ‘I knew nothing about stigma so I learnt that it is when people call other people with HIV with all sorts of bad names and that is painful … ’ They became skilled in helping PLWH cope with HIV stigma: ‘ … learnt how to cope and how to deal with the stigma’; ‘how to treat people with HIV and how to make them comfortable with their status’.

Friends felt proud of being a part of the projects and sharing the knowledge they had gained. ‘I feel proud and happy teaching something that is important;’ ‘We were able to teach others’; ‘Very proud and also that I still hold what I've taught and I am able to give people information.’

Friends from the rural group, like the group of family members, specifically noted the discriminatory acts within the healthcare setting. The rural group of friends, like the family members, indicated that they disapproved of the issue of colour coding the files of PLWH: ‘Because some of the people are faced with difficulty when they have to get treatment;’ ‘ … if they carry those black files, people know that they are HIV positive. And they don't want people to know … ’

One of their objectives planned initially was to address this, but this could not succeed due to refusal by the hospital authorities: ‘And the management said that it was not possible. We can write to the district, they give us the address. They said we can write to the district manager to ask them to change the files’. They felt that HIV stigma reduction was imperative and should continue in the community: ‘Because this workshop were very important for other people outside so they will teach us how to make an organisation so that we can help the people outside.’ They felt that standing together in unity against stigma in the community was possible: ‘I share that if we can work together, we can reduce this stigma thing.’

### 6.6. Experiences of spiritual leaders

During the workshops, spiritual leaders gained knowledge that brought them to a shocking reality of their role in applying the knowledge they had gained on HIV stigma: ‘I'm supposed to take the knowledge to be a spiritual leader or pastor and put it aside and also use this knowledge of stigma.’ They described coming to the realisation of how wrong they had been in the past in ignoring the issue of HIV stigma in their communities: ‘In the past … I am a pastor, these things are not my problem’; ‘I was taught in my college to read the Bible and telling the people do that and that, singing and praying then other things they not for me.’

They were brought to realise the negative impact stigma has on PLWH and that, as people with a standing in their community, their active involvement in stigma reduction was undeniable: ‘I am supposed to take the knowledge to be spiritual leader or pastor and put it aside and use this knowledge of stigma;’ ‘I am supposed to be taking out and reach out to the people and giving what I have.’

Spiritual leaders indicated that it had initially been a strange yet fulfilling experience to be placed together in a group of people from different religious and traditional standpoints. However, they realised that they were there for a common goal, wanting to get a better understanding of HIV stigma: ‘ … pleased to see us all as traditional healers as well as pastors being grouped together’; ‘working together especially people who are different in religions is totally something very difficult … but what I like is that we're serving one goal.’

The rural group of spiritual leaders was proud that people had gone for HIV testing spontaneously during the project hosted by them: ‘ … were willing to get tested because they used to fear being tested but they did at that time;’ ‘ … and those that were afraid to go and test ended up having the courage to go … ’ They further expressed a sense of pride and motivation to continue with the important task of HIV stigma reduction: ‘I saw what we were doing was something very good and important … our community is still happy because they ask us when next are you coming;’ ‘ … very excited to learn about stigma so that I could be able to explain it to my peers as well as the community.’

The realisation of being of support to PLWH was marked: ‘They feel free because people accepted them the way they are;’ ‘I can lead the people who are living with HIV and also those who do not have HIV.’ One of the spiritual leaders from the urban group felt able to disclose his own HIV status during the intervention due to other participants doing that comfortably within the group: ‘I met other spiritual leaders, the community. They made me stronger because I was able to disclose.’ They saw the success story by working together: ‘ … after the drama the whole thing was a success. So if everyone works together we will achieve.’

### 6.7. Experiences of community members

The community members described the intervention as initially frightening due to its unfamiliar nature, yet significant: ‘ … hard because I saw new faces that I didn't know but eventually I got used to them and we went and got taught and at the workshop I learnt things that I didn't know.’ They received knowledge about stigma and coping with it and realised the importance of sharing the knowledge with others: ‘ … knowledge I gained from the workshop like HIV stigma, coping … ;’ ‘ … take this information that I had and use it in my community with my friends and share it with my people and my peers.’

They were able to work in close proximity with PLWH during the intervention and realised that they were equal to everyone else: ‘I was able to deal with and working with people who are positive. I was able to put myself in their shoes;’ ‘I didn't see them as the outsiders. I started to see them as people just like us.’ During the intervention, the community members became aware that dedication is necessary for a project to be successful: ‘Even if you must stay up the whole night, it doesn't matter because it is you that is going to have the fruits behind it. You will get the fruits.’ The projects were successful and well received in the communities: ‘It was so fruitful because the impact we get from the people were very good;’ ‘Even though it was a good thing, it was a success for me.’ They were also able to realise how important and satisfactory it was to share gained knowledge: ‘I can also help some people gaining knowledge because with the little they gain we can at least do something more in helping the next person.’

Community members expressed feelings of motivation after the intervention. Some felt inspired by the passion from presenters to also step out and act against stigma: ‘I can see that these people are really serious and these people they are not here to play these people are here to help others;’ ‘ … I'm going to join them and I'm going to help also to make a difference.’

## 7. Conclusions

There were no remarkable differences between the experiences expressed by the PLWH and the people living close to them of the urban and rural groups after they had undergone the comprehensive HIV stigma reduction community intervention. This could be due to the Setswana culture that is prominent in the North West Province. After they had taken part in the intervention in its totality with the designated groups of people living close to them, the PLWH expressed a sense of being accepted and respected. Bringing the people together had made them aware that they still had human dignity, and that they could still live positively with the virus.

The workshop was described as emotionally demanding mainly due to the imminent disclosure, but support and the eventual disclosure actually brought freedom for PLWH. Responsible disclosure management was discussed and truly capacitated PLWH for the management of HIV disclosure in a responsible manner.

They received knowledge about HIV stigmatisation as well as skills for coping with it. They felt enabled to actively take part in HIV stigma reduction in the community. The PLWH also reported that designing a project as part of the intervention had been difficult, although it had proved to be successful in the end and had instilled a sense of pride. The PLWH strongly agreed that they wished to reach many people with the important message of HIV stigma reduction.

Partners gained knowledge on HIV stigma, but they reported a strong realisation of their stigmatising behaviour towards PLWH. They could accept themselves better. Relationships between partners were enhanced during the intervention. Disclosures that took place during the workshop as part of the intervention empowered some partners to also take that step. The knowledge gained empowered them to share their status and also to change their stigmatising behaviour. The partners experienced the intervention as an uplifting and enriching experience.

Children were initially nervous when they started the intervention, but they learned that it was an enriching experience and they gained confidence. They were saddened by the severity of HIV stigmatisation and they became aware of their damaging deeds as participants in stigmatisation. There was a common goal amongst them to advocate against HIV stigmatisation and a strong sense of cohesion was reported. The children gained self-confidence with the success of their projects.

The family members reported gaining an enhanced understanding of HIV stigma. The prominent theme reported by family members was the positive change in the attitudes of family members brought about by the intervention. Leadership was enhanced and HIV stigma activists formed. The rural group of family members experienced noticeable discriminatory acts within the healthcare environment. The projects were experienced as difficult but meaningful.

The group of friends experienced gaining knowledge regarding HIV stigma, but they prominently indicated the realisation of the painful effects of stigma. Initially there was a strong sense of fear of contagion in this group, but that changed. They also reported gaining skills on how to help PLWH cope with stigmatisation. They further felt proud of the useful knowledge received and their being able to share it. The rural group of friends, like the family members, reported their dismay at realising the visible discrimination in the healthcare settings where PLWH were being marginalised by the existing filing system. The friends uniformly described their strong belief in the importance of applying HIV stigma reduction in a continuous and sustainable manner in the community.

Spiritual leaders realised that HIV stigma knowledge was important and should be applied in their work as spiritual leaders. They acknowledged their negligent behaviour in the past with regard to HIV stigma reduction and also that they had the capacity as respected figures in the community to assist in HIV stigma reduction. They reported that being placed in a group of people with varying religious and traditional viewpoints was strange and challenging, but eventually they worked towards a common goal of HIV stigma reduction and recognised how fulfilling the experience actually was. It made them to feel proud of being part of the project and motivated to reduce HIV stigma in the community. There was a strong sense of being a support system to PLWH. The spiritual leaders acknowledged the importance of working together to achieve set goals.

Community members experienced the workshops as being frightening due to the unknown nature thereof, but they eventually found them meaningful. They gained knowledge about HIV stigma and coping with it and they reported the strong realisation of the importance of sharing gained knowledge. The community members were reminded that PLWH were equal to any other human being and that they should not be treated differently. They also realised that dedication was an important aspect of ensuring success in a project. They felt motivated to go out and assist in HIV stigma reduction in their community.

From the discussion of the results, it can be concluded that not only was there an increase in knowledge, but also all designated groups as well as the PLWH reported an experience of enhanced relationships and being equipped with leadership skills in order to go out into the community and being part of HIV stigma reduction actions. However, the PLWH reported more prominently on being enlightened about being accepted and that they were able to live a normal life in a community with PLWH as well as non-infected individuals. Responsible disclosure management was identified as a very meaningful guideline for future disclosure decisions.

Partners realised what a major role they were playing in stigmatising PLWH and they were reminded of the fact that PLWH should be treated with respect and dignity. Children commonly reported on a sense of enhanced relationships and cohesion with other children in the group. They further became aware of how painful HIV stigmatisation is for these children. They became motivated and actively took part in HIV stigma reduction efforts in the community.

In the group of family members, there was a uniform report that the intervention had brought about changes in negative attitudes of family members towards PLWH. Friends reported on their realisation of how prominent their role in HIV stigmatisation was. They often feared contagion unnecessarily and they became aware of the pain that they were causing by their stigmatising behaviour.

Spiritual leaders noted that they had been negligent in the past with regard to HIV stigma reduction by saying that it was not part of their duty as spiritual leaders to assist in HIV stigma reduction. However, they understood the important role they could play in HIV stigma reduction, as it was a prominent problem in the community, and that they as figures carrying authority in the community could make a significant difference in fighting HIV stigma.

This intervention involving PLWH and the several designated groups as a whole fills a gap in the knowledge field of stigma interventions.

## 8. Limitations

Some of the designated group members' groups were smaller than anticipated because the PLWH had a choice to bring a specific member of a designated group, or they did not have someone who represented the designated group, e.g. a partner or child.

## 9. Recommendations

Although the intervention in its entirety was successful after it had been implemented, it could be meaningful to take the specific needs of the different groups involved in this study into consideration, as a greater awareness of these needs was created. With regard to partners, it may be meaningful to also specifically address HIV stigmatisation in the context of the PLWH being in intimate relationships with the partners.

Children may gain from preparing them emotionally to be part of a group of PLWH who may be older than they are. Family members can be placed together with more family members from the same family to intensify the effect of the intervention on more people. Friends in this study seemed to have been strongly taking part in stigmatising behaviour, and fear of contagion remained an issue. It may be worthwhile bringing friends together and providing them with knowledge on HIV modes of transmission and advocating friendships with PLWH, as they are the same as anyone else.

Spiritual leaders could be specifically trained on HIV stigma and the religious community, e.g. on the application of biblical norms in HIV stigma reduction education. Applying the intervention in its totality with the projects is effective, as many people were eventually reached due to its ripple effect. Further quantitative research to strengthen the findings of this study could be meaningful – also to evaluate its long-term effect. It can also be useful to take the intervention further into the community on a permanent basis, for example by establishing HIV stigma reduction hubs in the broader community. Additional research involving other cultural groups and urban and rural populations would be meaningful.

The effect the intervention had on both the urban and the rural group makes it especially useful for other parts of Africa as well.

Using the intervention as it is with all the designated groups is the ideal, but could because of its length and time intensity become costly. Instead the PLWH could be asked to bring any one person of their choice living close to them and have a mixed group of people living close to them during the intervention.
